# The impact of a fellow-driven debriefing program after pediatric cardiac arrests

**DOI:** 10.1186/s12909-019-1711-y

**Published:** 2019-07-22

**Authors:** Jennifer Gillen, Monica L. Koncicki, Rebecca F. Hough, Kathryn Palumbo, Tarif Choudhury, Ariel Daube, Anita Patel, Amy Chirico, Cheryl Lin, Sirisha Yalamanchi, Linda Aponte-Patel, Anita I. Sen

**Affiliations:** 1 NewYork-Presbyterian Morgan Stanley Children’s Hospital, New York, NY USA; 2 Present affiliation: Kravis Children’s Hospital, Mount Sinai Medical Center, New York, NY USA; 3 Present affiliation: St. Christopher’s Hospital for Children, Philadelphia, PA USA; 4 Department of Pediatrics, Columbia University Medical Center, New York, NY USA; 5 Present affiliation: Maimonides Medical Center, Brooklyn, NY USA; 6 Present affiliation: Children’s National Medical Center, Washington, DC USA; 7 Present affiliation: Rutgers University – Robert Wood Johnson Medical School, New Brunswick, NJ USA; 8 Pediatric Critical Care Medicine, NewYork-Presbyterian Morgan Stanley Children’s Hospital, 3959 Broadway CHN 10-24, New York, NY 10032 USA

## Abstract

**Background:**

In the United States, post-cardiac arrest debriefing has increased, but historically it has occurred rarely in our pediatric intensive care unit (PICU). A fellow-led debriefing tool was developed as a tool for fellow development, as well as to enhance communication amongst a multidisciplinary team.

**Methods:**

A curriculum and debriefing tool for fellow facilitators was developed and introduced in a 41-bed cardiac and medical PICU. Pre- and post-intervention surveys were sent to multidisciplinary PICU providers to assess effectiveness of debriefings using newly-trained leaders, as well as changes in team communication.

**Results:**

Debriefing occurred after 84% (63/75) of cardiac arrests post-intervention. Providers in various team roles participated in pre-intervention (129 respondents/236 invitations) and post-intervention (96 respondents /232 invitations) surveys. Providers reported that frequently occurring debriefings increased from 9 to 58%, pre- and post-intervention respectively (*p* < .0001). Providers reported frequent identification and discussion of learning points increased from 32% pre- to 63% post-intervention. In the 12 months post-intervention, 62% of providers agreed that the overall quality of communication during arrests had improved, and 61% would be more likely to request a debriefing after cardiac arrest.

**Conclusion:**

The introduction of a fellow-led debriefing tool resulted in regularly performed debriefings after arrests. Despite post-intervention debriefings being led by newly-trained facilitators, the majority of PICU staff expressed satisfaction with the quality of debriefing and improvement in communication during arrests, suggesting that fellow facilitators can be effective debrief leaders.

## Background

Since high quality cardiopulmonary resuscitation has been shown to improve return of spontaneous circulation and survival rates after arrests, strategies to improve resuscitation quality have become important areas of research [1–3]. Structured, post-event debriefing has been shown to improve team performance in military and aviation training, where high-stress but infrequent events occur, similar to the hospital environment [4–6]. As a result, many hospitals are integrating formal debriefing after cardiac arrest into routine care, often with attending physicians in leadership roles [5]. Developing a curriculum to train and allow physician-trainees and front-line staff to facilitate debriefings after cardiac arrests is potentially a valuable strategy for both faculty development and for enhancement of multidisciplinary team communication.

Structured debriefings have been shown to increase the feasibility of introducing a debriefing program [7, 8]. Specifically for novice facilitators, a scripted structure has been shown to improve the efficacy and standardization of debriefing after simulation [9]. As a result, scripted debriefing has been used in the Pediatric Advanced Life Support (PALS) training course after simulated arrests since 2009 [10]. Most pediatric providers are PALS certified and thus have had experience with such structured debriefing in simulation environments. In an effort to create a debriefing tool familiar to and easy for pediatric critical care fellow facilitators to use, the PALS script for debriefing after cardiac arrest was adapted. The goals of this study were to 1) develop a debriefing tool and debriefing training curriculum, 2) train fellows to use the debriefing tool, 3) implement a standardized debriefing after each cardiac arrest in the PICU using fellow facilitators, 4) determine the effectiveness of fellow-led debriefings and 5) assess the effect of fellow-led debriefings on communication amongst multidisciplinary team providers.

## Methods

**Fig. 1:**
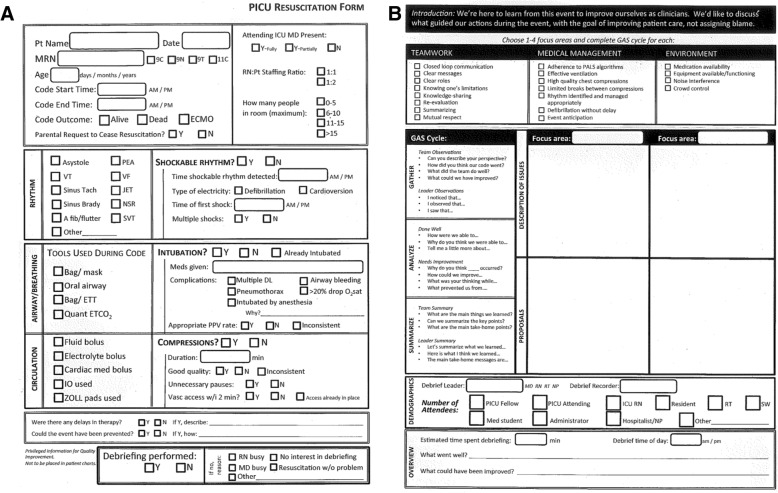
Cardiac Arrest Data Collection Forms; **a**: Cardiac Arrest Data Sheet **b**: Cardiac Arrest Debriefing Tool

To better identify targets for improvement in teamwork and medical management of life-threatening events occurring in the PICU, we designed a fellow-driven debriefing program. The main intervention for this project was the creation and implementation of two tools to standardize debriefing: the Data Sheet and the Debriefing Tool. The Data Sheet (Fig. 1a) created a detailed record of each event (i.e. time of event, length of compressions, need for intubation, etc.) to aid in analysis of such events at a later date. The Debriefing Tool (Fig. 1b) was created to provide guidance for post-arrest debriefing using a modified version of the Pediatric Advanced Life Support debriefing script [10]. Cardiac arrest was defined as an event in the PICU requiring one or more of the following: 1) greater than 1 minute of chest compressions, 2) defibrillation, or 3) unstable rhythm requiring cardioversion. If an event did not fulfill the time requirement for cardiac arrest but was deemed clinically significant by the fellows, it was included in the arrest list for analysis. Events requiring cardioversion were included in this study as they are rare pediatric events worthy of review.

PICU fellows, as they consistently attend all arrests in the PICU where attendings are not overnight in house, were selected as the formal debriefing leaders. The fellows were introduced to the Data Sheet and Debriefing Tool during fellow orientation. Fellows also attended a yearly faculty-led debriefing workshop to refresh debriefing skills. The workshop included simulated scenarios and role-playing as debrief leaders, as well as orientation to the Debriefing Tool.

The study of this intervention took place at an urban, quaternary care hospital with a 41 bed PICU, caring for all critically-ill pediatric medical, surgical and cardiac patients (excluding those in the neonatal intensive care unit). Debriefing was expected after every arrest, occurred in the PICU team room, and, whenever possible, occurred within the same nursing shift as the event. All resuscitation team members were invited to participate in debriefings (i.e. fellows, attendings, nurses, residents, nurse practitioners, hospitalists, respiratory therapists, social workers, consultants, administrators, etc.).

The debriefing facilitator, typically the fellow who had participated in the arrest, began by welcoming the participants, followed by an assurance that the discussion was occurring without judgment and that the goal was to improve team resuscitation skills. The facilitator next asked the group for general thoughts about the resuscitation. Finally, the facilitator selected two to three focus areas (topic suggestions listed at the top of the Debriefing Tool) and led the team through Gather, Analyze, Summarize Cycles addressing each area. As the debriefings were intended to occur directly after the arrests, the debriefing was not data-driven. Discussion points were recorded in the Debriefing Tool for later analysis. For the purpose of this study, topics most frequently discussed during the debriefings were analyzed using the Team Emergency Assessment Measure (TEAM) framework, a validated tool to evaluate medical resuscitation [11]. The Data Sheet was completed by the facilitator and PICU charge nurse after the debriefing.

**Fig. 2:**
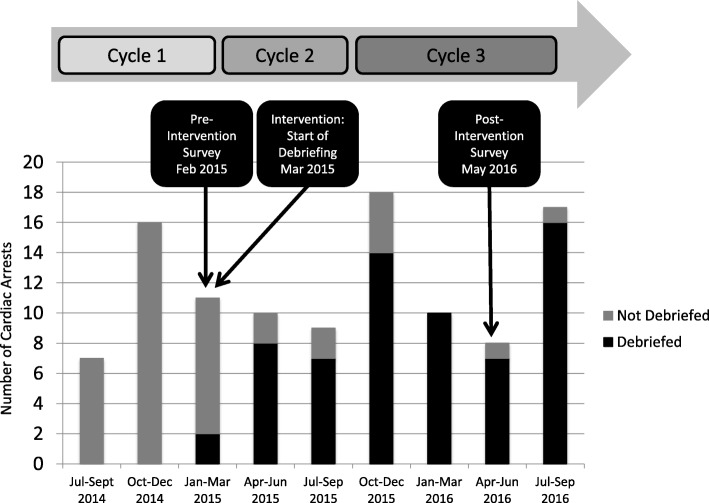
Project Timeline, Frequency of Cardiac Arrest, and Debriefing Frequency. PDSA Cycle 1 (Jul 2014 – Feb 2015): Initiation of cardiac arrest data collection, creation of cardiac arrest data sheet and debriefing tool. PDSA Cycle 2 (Mar 2015 – Sep 2015): Initiation and revision of arrest data sheet and debriefing tool. PDSA Cycle 3 (Oct 2015 – Sep 2016): Implementation of finalized arrest data sheet and debriefing tool

Through a series of Plan-Do-Study-Act (PDSA) cycles (Fig. 2), the Data Sheet and Debriefing Tool were created and revised in PDSA Cycle 1 (July 2014 to February 2015) using feedback and suggested improvements from PICU staff. Revisions were minor and included edits to data collection on the Data Sheet, as well as additions of focus area topics on the Debriefing Tool. PDSA Cycle 2 (March 2015 to September 2015) included the implementation of the major intervention of fellow-led debriefing, as well as ongoing revision of the Data Sheet and Debriefing Tool (primarily formatting changes). PDSA Cycle 3 (October 2015 to September 2016) comprised the implementation of the finalized Data Sheet and Debriefing Tool (Fig. 1a and b).

Faculty provided monthly feedback to the PICU fellows regarding their compliance with debriefing and use of the Debriefing Tool. Additionally, a survey (consisting of 24 multiple-choice questions and one free-text response) was emailed to PICU providers in February 2015 (pre-intervention) and May 2016 (post-intervention) to gather data from multidisciplinary team members (attending physicians, fellows, hospitalists, nurse practitioners, pediatric residents, respiratory therapists, and PICU nurses) regarding their attitudes and experiences surrounding debriefing and communication. Participation in the surveys was voluntary and anonymous and conducted via an online website (SurveyMonkey.com). After completing the survey, staff members were invited to enter a voluntary raffle for a $20 gift card. Responses to survey questions were analyzed. Descriptive statistics were used to analyze the quantitative data from arrest sheets; categorical questionnaire responses were analyzed using 2-tailed Fisher’s exact tests. A *p* value of < 0.05 was considered significant.

## Results

During the 27 months of this study, 105 cardiac arrests were documented; 75 arrest Data Sheets and 63 formal debriefings using the Debriefing Tool were completed (Fig. 2). Post-intervention, the rate of debriefing after cardiac arrest was 84%. Throughout the post-intervention study period, debriefing rates increased from 75% over the first 10 months to 94% over the final 9 months.

Detailed characteristics of the cardiopulmonary arrests from the study period are described in Table 1. All 75 arrests in the post-intervention period contained chest compressions of at least 1 minute or greater. Topics most frequently discussed during the 63 debriefings performed included: communication 67%; cooperation/coordination 59%; other-equipment 51%; clinical standards 38%; situational awareness 27%; other-extra-corporeal membrane oxygenation (ECMO) 21%; team climate 16%; leadership 13%; adaptability 3%; prioritization 0% (percentages total more than 100 as multiple topics were discussed in individual debriefings). For the twelve arrests after which no debriefing occurred in the post-intervention period, the reasons given were as follows: nurse and/or physician too busy 58%; physician too busy 8%, change of shift 25%; another patient decompensating 8%; no reason provided 17% (percentages total more than 100 as multiple answers were allowed).

**Table 1:** Description of Post-Intervention Cardiac Arrest Characteristics

Cardiac Arrest Characteristic	Post-Intervention Cardiac Arrests n (%)
Age, years^a^	1.9 (0.5–7.0)
Gender, Male	36 (48)
Time of Day	
08:00–15:59	29 (39)
16:00–11:59	20 (27)
00:00–07:59	26 (35)
Outcome	
Dead	24 (32)
Alive	34 (45)
ECMO	17 (23)
Cardiac Disease	42 (56)
Attending Present at Arrest (*n* = 69)	58 (84)
Nurse to Patient Ratio (*n* = 65)	
1:1	51 (78)
1:2	14 (22)
Maximum Arrest Team Size (*n* = 64)	
≤5	6 (9)
6–10	36 (56)
11–14	14 (22)
≥15	8 (13)
Airway Placed During Arrest (*n* = 73)	
Yes	21 (29)
Already in place	51 (70)
No	1 (1)
Duration of CPR, min.^a^ (*n* = 74)	11 (4–39)
Defibrillation during CPR	11 (15)
Duration of Debriefing, min.^a^ (*n* = 57)	20 (15–20)
Delays in Care (*n* = 61)	21 (34)
Preventable Event (*n* = 58)	7 (12)

A total of seventy-five cardiac arrests were analyzed. The arrest characteristics with incomplete data sets are indicated above with specific n values

^a^Median (IQR)

Overall survey response rate was 55% (129 respondents out of 236 invitations) for the pre-intervention survey and 41% (96 respondents out of 232 invitations) for the post-intervention survey. The responder role did not differ significantly between the two surveys (Table 2). Pre-intervention response rates by team member role were (respondents/invitations): attending physicians (11/17), fellows (5/9), nurse practitioners/hospitalists (7/10), pediatric residents (21/49), respiratory therapists (7/21) and registered nurses (78/130). Post-intervention response rates were (respondents/invitations): attending physicians (12/18), fellows (5/10), nurse practitioners/hospitalists (7/18), pediatric residents (17/50), respiratory therapists (10/18) and registered nurses (45/118). There was a significant increase in satisfaction with debriefing, identification of learning points during debriefing, and the belief that debriefing after arrests should be standard. Over half (52%) of the providers in the pre-intervention survey group reported experience with “more than 10” resuscitations in our PICU versus 43% of the post-intervention survey group (*p* = 0.18).

**Table 2:** Pre- and Post-Intervention Survey Responses

Survey Item	Pre-Intervention (%)	Post-Intervention (%)	p value
My role in the PICU is:			
*PICU Attending/Fellow*	16 (11)	17 (16)	
*Hospitalist/Nurse Practitioner*	7 (5)	7 (7)	
*Pediatric Resident*	37 (26)	26 (25)	
*Respiratory Therapist*	7 (5)	10 (10)	
*PICU/PCICU Nurse*	78 (54)	45 (43)	
I (*strongly agree/agree*) it is important to have a formal debriefing after an arrest	111 (79)	86 (90)	0.05
I (*disagree/strongly disagree*) that I would feel uncomfortable participating in a debriefing after an arrest	107 (78)	84 (88)	0.08
I (*strongly agree/agree*) that I am satisfied with the amount of debriefing I’ve experienced after arrests	18 (14)	55 (57)	< 0.01
When I have observed debriefings following an arrest, specific learning points were identified and discussed during the debriefing (*all of the time/frequently*)	43 (32)	60 (62)	< 0.01
When I have observed debriefings following an arrest, specific learning points were subsequently disseminated to the rest of the PICU staff who did not participate in the arrest (*all of the time/frequently*)	5 (4)	11 (11)	0.03
I (*strongly agree/agree*) the experience of debriefing has resulted in potential changes to my practice for future arrests	60 (46)	64 (67)	< 0.01
I (*strongly agree/agree*) debriefing after arrests should be standard practice	96 (73)	91 (95)	< 0.01

The number of responders to each individual question varied. Pre-Intervention Survey n range: 131 to 145; Post-Intervention Survey n range: 95 to 105

In addition to an actual increase in prospectively measured frequency of debriefing, the perceived frequency of debriefing (as assessed by multidisciplinary PICU staff survey) also increased post-intervention. Perceived debriefings occurring at least frequently increased from 9 to 58%, pre- and post-intervention respectively (*p* < 0.0001). The most frequently reported debriefing leader pre-intervention was the PICU attending (45%), whereas post-intervention, the PICU fellow (71%) was reported as the most frequent debriefing leader. Additional questions in the post-intervention survey assessed changes in provider practice related to debriefings (Table 3). Of providers who had participated in a debriefing in within the prior year pre-intervention, 69% felt that the quality of debriefing had improved and 73% were satisfied with the quality of debriefing post-intervention. In the twelve months post-intervention, 62% of providers agreed that the overall quality of communication during arrests had improved, and 58% agreed that interdisciplinary team interactions had improved. Post-intervention, 61% of providers agreed that they were now more likely to request a debriefing after cardiac arrest. Thirty-eight percent of respondents agreed that adherence to PALS guidelines during an arrest in the year post-intervention had improved.

**Table 3:** Post-Intervention Survey Assessment

Providers Felt:	Strongly Agree/ Agree	Neutral	Disagree/ Strongly Disagree
Satisfied with quality of debriefing^a^	73%	20%	8%
Overall communication during an arrest had improved in the past year	62%	34%	3%
Interdisciplinary interactions during an arrest had improved in the past year	58%	40%	2%
Adherence to PALS guidelines during an arrest had improved in the past year	38%	57%	5%
He/she would be more likely to ask for a debriefing if one had not been initiated	61%	28%	10%

^a^Excludes participants who had never observed a debriefing (*n* = 87)

## Discussion

Debriefing is a guided conversation among team members, with the goal of encouraging reflection on team performance [8, 12]. This reflection has been shown to be a critical step in experiential learning as it aids in identifying changes in practice for future events [13–15]. Previous literature describes senior physicians (often attending physicians removed from the actual event) leading debriefings [16], with more recent literature exploring other team members taking on this role [17]. Overall, there is a lack of literature describing how to best approach faculty development programs regarding debriefing [18]. To our knowledge, this is the first debriefing program described in the literature targeting trainees as facilitators.

For facilitators without significant debriefing experience, a formalized script improves the standardization of debriefing [9]. The PALS course has used such scripted debriefings in training courses with the goal of improving patient care practices [10]. As debriefing after simulation has been shown to improve teamwork skills [19], the PALS script was modified in our curriculum development, used in training sessions, and ultimately, used to guide debriefings. Over two-thirds of our PICU staff members felt that fellow-led, scripted debriefing had improved the quality of debriefing in our unit. There were perceived improvements in communication, interdisciplinary interactions, adherence to PALS guidelines and overall comfort asking for a debriefing. Using this curriculum to transition from an attending-led to a fellow-led debriefing program demonstrated the development of key leadership skills in fellows, as well as a sense of better communication amongst interdisciplinary team members, with minimal time and cost invested.

Our PICU overcame the common barriers to developing a culture of debriefing, including a lack of trained facilitators, standardized process and uniform debriefing content [17, 20–22]. Debriefing is now the expected process: post-intervention debriefings occurred at a rate of 84%. This rate compares favorably to the debriefing rate of 47% reported in a recently published international study [22]. We have demonstrated that given the proper support (a curriculum and scripted debriefing tool), clinicians other than attending physicians can be trained as effective debriefing leaders.

Literature describing debriefing development programs is sparse [18], but the potential for trainee development is clear. Having a fellow-led debriefing program can fulfill Accreditation Counsel for Graduate Medical Education (ACMGE) requirements, develop fellows’ skills as clinician-educators, and address trainee core competency requirements [21]. Additionally, adapting scripts for debriefing leaders could be applied to other clinicians, such as nurses or residents, with similar benefits. A recent study showed that using a simple debriefing tool allowed charge nurses in the emergency room to take the lead as debriefing leaders, promoting a culture of debriefing [17]. Similarly, debriefing can be helpful for events other than cardiopulmonary arrests, such as interactions with difficult families, breaking “bad news” or unexpected events during routine procedures [21]; its use extends far beyond the intensive care unit and emergency room.

Debriefing can take many forms, with the optimal form yet to be determined. This study was based upon a “hot debriefing” model [6, 22]. Hot debriefing occurs immediately after an event and is centered on the qualitative reactions of the resuscitation participants [6], with the benefit of having the actual resuscitation team members present. As these debriefings immediately follow an arrest, objective data from defibrillators and monitors are not typically analyzed prior to the debriefing [6]. However, despite this, important qualitative information can be obtained from hot debriefings beyond actual CPR mechanics, including concerns of treatment delay, suboptimal leadership and equipment concerns [6, 22]. Our study found that our fellow-led hot debriefing program resulted in not only trainee leadership development, but also improved perception of team communication and the identification of experiential learning points that led to changes in clinical practice in our PICU. These changes focused on the anticipation of arrest events, identification of clear roles during resuscitation, avoidance of equipment malfunctions, and improvement of teamwork. Although developing quality improvement projects was not a primary objective of this study, quality initiatives emerged from the introduction of hot debriefing, including an “Arrest Phone Tree” to recruit key personnel to the arrest, code role stickers to limit overcrowding, and an ECMO initiation checklist to ease pre-cannulation management. Evaluation of the effectiveness of these interventions on care during resuscitation is ongoing.

In contrast, “cold debriefing” occurs days, weeks, or months after an event, and includes objective, analyzed data about the quality of resuscitation that can then be disseminated to all team members, even those not present at the arrest [4]. Cold debriefing has been shown to improve the quality of resuscitation and outcomes [4, 5], but requires significantly more preparatory work to function. Cold debriefings allow for data-driven analysis of resuscitation areas such as CPR quality, which are known to be inaccurately recalled by providers [23]. We acknowledge the value of such data-driven debriefing, but we believe that both hot and cold debriefings can be beneficial. For our institution, changing the debriefing culture by instituting fellow-led hot debriefing allowed us to capture immediate feedback from resuscitation team members, which then allowed us to make targeted improvements to our resuscitation approach.

This study includes several limitations. First, the pre- and post-intervention survey relied on voluntary email responses and post-intervention response rates were comparatively low. This decrease in response rate was likely related to high nursing turnover during the study period, as nurses were the largest overall responder group. Response rate also may have been influenced by selection bias, with participants who approved of the debriefing model more likely to respond to the survey, thus overestimating its positive impact. Second, the debriefing tool and curriculum was not specifically designed to address the emotional support needs of the staff, which typically manifested as a major focus area for debriefing. However, despite not focusing on this aspect during the design, it was described anecdotally that the debriefings were helpful to relieve stress after critical events. Third, we did not attempt to quantitatively measure the frequency of debriefing pre-intervention, instead relying on both staff survey results and personal experience to measure pre-intervention debriefing frequency; debriefing pre-intervention was uncommon and erratic in our PICU. Fourth, although staff satisfaction and comfort levels were measured, the intention of this project was not to discern an effect on mortality. Although perceived adherence to PALS increased and there were quantitative trends towards better adherence to standard practices (i.e. more consistent use of end tidal monitoring), detecting a change in mortality rate would likely not be feasible due to the historically low mortality rate in our PICU. Finally, although there was an increase in learning point identification, the perceived frequency of learning point dissemination remained low post-intervention. Improving the dissemination of learning points from debriefing will be the focus of ongoing quality improvement efforts.

## Conclusion

The development and implication of a debriefing curriculum may be an effective tool in leadership development for fellow facilitators, as well as a means to improve communication amongst interdisciplinary team members in the PICU. The simplicity and low cost of adapting scripts for fellow debriefing leaders could be applied to other clinicians, such as nurses or residents, and to events other than cardiac arrest; its use may extend far beyond the intensive care unit. Given the proper support in the form of a scripted debrief tool, fellow facilitators develop valuable leadership skills and may provide a means to improve teamwork and communication throughout the hospital.

## Data Availability

Consent was waved, as above. Consent from survey participants for data dissemination beyond the purposes of this study was not obtained. Therefore, specific data from this project cannot be shared.

## Abbreviations

ACGME: Accreditation Council for Graduate Medical Education
CPR: cardiopulmonary resuscitation
ECMO: extracorporeal membrane oxygenation
IQR: Interquartile Range
PALS: Pediatric Advanced Life Support
PDSA: plan – do – study – act
PICU: pediatric intensive care unit
TEAM: Team Emergency Assessment Measure
